# Essential transcription factors for induced neuron differentiation

**DOI:** 10.1038/s41467-023-43602-7

**Published:** 2023-12-15

**Authors:** Congyi Lu, Görkem Garipler, Chao Dai, Timothy Roush, Jose Salome-Correa, Alex Martin, Noa Liscovitch-Brauer, Esteban O. Mazzoni, Neville E. Sanjana

**Affiliations:** 1 New York Genome Center, New York, NY USA; 2 Department of Biology, New York University, New York, NY USA; 3 Present Address: Department of Cell Biology, NYU Grossman School of Medicine, New York, NY USA

**Keywords:** Functional genomics, Development of the nervous system, Stem-cell differentiation

## Abstract

Neurogenins are proneural transcription factors required to specify neuronal identity. Their overexpression in human pluripotent stem cells rapidly produces cortical-like neurons with spiking activity and, because of this, they have been widely adopted for human neuron disease models. However, we do not fully understand the key downstream regulatory effectors responsible for driving neural differentiation. Here, using inducible expression of *NEUROG1* and *NEUROG2*, we identify transcription factors (TFs) required for directed neuronal differentiation by combining expression and chromatin accessibility analyses with a pooled in vitro CRISPR-Cas9 screen targeting all ~1900 TFs in the human genome. The loss of one of these essential TFs (*ZBTB18*) yields few MAP2-positive neurons. Differentiated *ZBTB18*-null cells have radically altered gene expression, leading to cytoskeletal defects and stunted neurites and spines. In addition to identifying key downstream TFs for neuronal differentiation, our work develops an integrative multi-omics and TFome-wide perturbation platform to rapidly characterize essential TFs for the differentiation of any human cell type.

## Abstract (web summary)

Using integrative multi-omics and CRISPR knock-out of all ~1,900 transcription factors, the authors identify essential transcription factors required for Neurogenin-driven differentiation of human cortical neurons.

## Introduction

During development, the temporal activation of transcription factors (TFs) in specific sequences generates the diverse neurons found in the human cortex^1^. Over the past two decades, multiple studies have demonstrated that the proneural TFs Neurogenin-1 (*NEUROG1*) and Neurogenin-2 (*NEUROG2*) are necessary and sufficient to specify glutamatergic neuronal identity^2–7^. These glutamatergic neurons populate the neocortex and other brain regions such as the hippocampus and olfactory bulb and comprise the primary excitatory network in the central nervous system^8–11^. *NEUROG1* and *NEUROG2* are expressed together in ~95% of cortical progenitors, although at slightly different times in development with NEUROG1 having an additional role of tempering the proneural effects of NEUROG2^12–14^.

In recent years, in vitro cell programming and differentiation studies have shown that glutamatergic neurons can be efficiently differentiated from human or mouse embryonic stem cells (hESCs) or induced pluripotent stem cells (hiPSCs) by overexpression of *NEUROG2* alone or together with *NEUROG1*^15–18^. The combination of *NEUROG2* overexpression with small molecule modulators of SMAD and WNT signaling act together to generate neurons with more mature electrophysiological properties, such as NMDA-mediated synaptic transmission^19^. As basic helix-loop-helix (bHLH) TFs, Neurogenins bind E-box motifs as either homodimers or heterodimers with the heterodimeric partner capable of finely-tuning cell identity^15,20,21^. Proneuronal bHLH TFs are usually transiently expressed, but often induce other bHLH factors in a “bHLH cascade”^2,22^. Several putative target genes of *NEUROG1* and *NEUROG2* have been identified using chromatin immunoprecipitation, subtractive hybridization, and reporter assays^15,22,23^, as well as silico mutation analysis of gene regulatory networks^9^. However, these studies suggest that a large fraction of the regulatory network downstream of proneuronal bHLH factors—such as essential TFs controlling neuronal features—is still not fully understood, likely due to their observational approach instead of direct genetic perturbation.

To identify essential TFs for neuronal differentiation downstream of Neurogenins, we developed a pooled CRISPR-Cas9 knockout screen targeting all transcription factors in human pluripotent stem cells and differentiated neurons using a knock-in MAP2-tdTomato reporter human embryonic stem cell line with doxycycline-inducible expression of *NEUROG1* and *NEUROG2* (*NEUROG1/2*)^24,25^. In parallel, we performed multiomic profiling of gene expression and chromatin accessibility to understand the temporal order of TF activation, enabling us to assemble directed gene networks of regulator and target TFs. By integrating these gene networks with the TFome-wide knockout screen, we identified key TFs whose loss significantly hinders neuronal differentiation. We clustered these essential neurogenic TFs by expression and assembled them within a hierarchical TF network. One of the top-ranked TFs from the CRISPR screen, ZBTB18 (also known as ZNF238, ZFP238 and RP58) is essential for complete NEUROG1/2-induced differentiation, similarly to its role in vivo (e.g. Xiang^26^, Xiang et al.^27^). ZBTB18 loss led to a large reduction in MAP2+ cells, resulting in immature neurons with severely stunted dendritic arborizations. Beyond neuron differentiation driven by *NEUROG1/2*, our study brings together TF-focused CRISPR screens and integrative multiomics (gene expression and chromatin accessibility over time) for the rapid determination of required TFs for differentiation of any cell type—a key step toward understanding cell regulatory networks and developing realistic human model systems and cell-based therapies.

## Results

### Transcription factor network inference in *NEUROG2/1-*induced neurons by integration of gene expression and chromatin accessibility

**Fig. 1:**
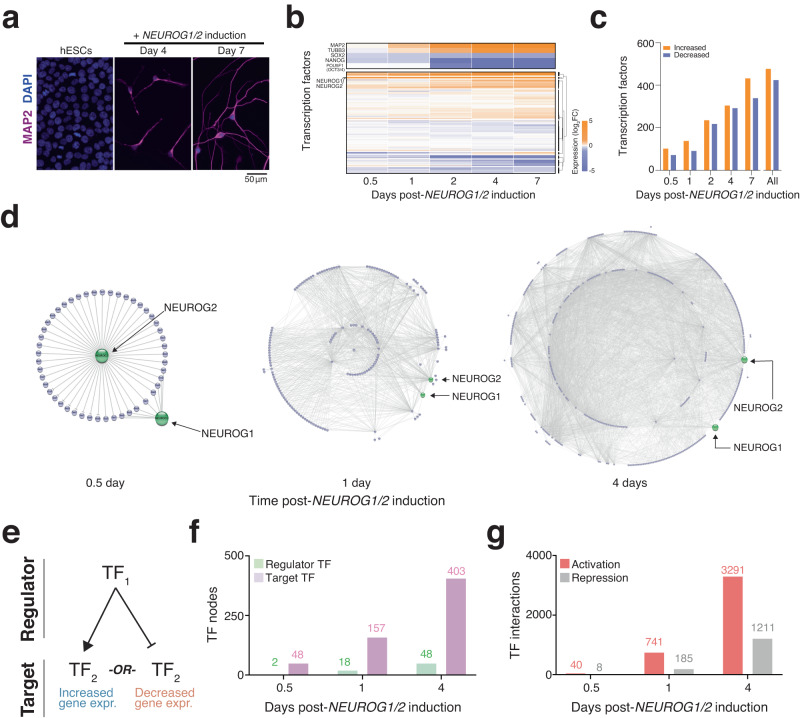
Integrating gene expression and chromatin accessibility to assemble transcription factor (TF) networks during the earliest stages of human neuron differentiation. **a** Representative immunofluorescence images of human embryonic stem cells and *NEUROG1/2*-induced human neurons stained with the neuronal marker microtubule-associated protein 2 (MAP2, purple) and the nuclear stain DAPI (blue). **b** Fold-change in the expression of all TFs over 5 timepoints during differentiation (*n* = 4 biological replicates at each time point). **c** Number of TFs with altered expression in developing iNs. **d** Network diagrams of TF at different timepoints after *NEUROG1/2* induction. Lines indicate a regulatory interaction between TF pairs. **e** Regulator TFs can act to either activate or repress their target TFs. **f** Number of regulators and target TFs at different timepoints after *NEUROG1/2* induction. **g** The number of activating and repressing TF interactions at different timepoints after *NEUROG1/2* induction.

We previously established NYGCe001 hESCs with doxycycline-inducible transcription factors *NEUROG1* and *NEUROG2* that we differentiated into induced neurons (iNs)^16,17,24^. As expected, iNs were MAP2-positive at Day 4 and displayed a neuronal morphology with long neurites by Day 7 (Fig. 1a). To determine the transcriptomic changes throughout neuron differentiation, we collected RNA from iNs at five timepoints (12 hours, 1 day, 2 days, 4 days and 7 days) and performed RNA-sequencing (Fig. 1b, Supplementary Data 1). As expected, neuron-specific genes like *MAP*2 and *TUBB3* were rapidly upregulated and pluripotency-associated TFs like *SOX2, NANOG* and *OCT3/4* were downregulated. Using the Allen Institute BrainSpan Atlas of Developmental Gene Expression, we compared gene expression in iNs to brain region-specific RNA-sequencing from 20 post-mortem donors using an average of 8 cortical regions per donor (Supplementary Fig. 1a, b). We found that differentially-expressed (DE) genes in Day 7 iNs are positively correlated with DE genes at early stages of fetal development (late first trimester and early second trimester) but not with DE genes at later stages (late second trimester and third trimester) (Supplementary Fig. 1c). Gene expression of in vitro iNs is most similar to early stages of fetal brain development (approximately 8–22 weeks post-conception), which corresponds to the peak period of neurogenesis with approximately 15 million neurons generated per hour^28^.

Out of the 1866 TFs previously cataloged in the human genome, we found 900 TFs with a more than 2-fold change in expression (*p*_*adj*_ < 0.05) during differentiation (Fig. 1c). The 900 TFs were near evenly split between genes with increased and decreased expression. Although *NEUROG1/2* act as transcriptional activators^3^, other TFs activated by the Neurogenin might act as repressors. Indeed, when classified by their DNA binding domains, differentially-expressed TFs span many different TF families that include both activators and repressors (Supplementary Fig. 1d).

We also performed ATAC-seq on the iNs at multiple time points after doxycycline induction (1 hour, 4 hours, 12 hours, 1 day, 5 days). We confirmed high correlation between biological replicates and fragment lengths with characteristic nucleosome banding (Supplementary Fig. 1d, e). We also verified an increase in open chromatin at the promoters of proneural genes over time (Supplementary Fig. 1f). To better characterize the connections between the pioneer TFs *NEUROG1/2* and the large number of differentially-expressed TFs, we constructed putative TF networks by combining data from chromatin accessibility, DNA binding motifs, and gene expression experiments. At different timepoints, we sought to connect regulator TFs to specific target TFs (Supplementary Fig. 1g). We identified putative regulators TFs as those expressed TFs with identified binding sites within the gene body (including the upstream promoter region) of putative target TFs. Our expression data indicated both activation and repression of TFs upon binding (Fig. 1c), so we wanted to capture both TF activation and repression in our analysis framework. Thus, we included target TFs with either a significant increase (activation) or significant decrease (repression). We measured chromatin accessibility changes genome-wide at several timepoints, starting as early as 1 hour after doxycycline induction. As a positive control, we examined all *NEUROG1* and *NEUROG2* motifs across the genome to characterize how accessibility at the binding sites of these pioneer TFs is modulated during differentiation. We observed minimal changes in accessibility at *NEUROG1* and *NEUROG2* motifs at 1 hour (*p*_*adj*_ > 0.05) and greater changes in accessibility starting at 12 hours post-induction (Supplementary Fig. 1h–i).

We next sought to connect regulator TFs to their target TFs by integrating RNA-sequencing and ATAC-sequencing data at 3 different time points during differentiation (12 hours, 1 day and 4 days) (Fig. 1d, e, Supplementary Data 1). At the first timepoint, we defined the regulator TFs as *NEUROG1* and *NEUROG2*—driven by doxycycline induction which occurred 12 hours earlier. To connect regulator TFs to putative target TFs, we used two independent criteria from accessibility and expression data. First, using ATAC-seq, we verified that the genomic locus of the target TF includes a predicted binding site of the regulator TF in an open chromatin region (see *Methods*). In addition, we required the target TF to undergo at least a 2-fold increase or decrease in expression (FDR *q* < 0.05). If both conditions are met (greater accessibility of regulator TF motif at target TF locus and altered expression of target TF), we connected the target TF to the regulator TF. In subsequent timepoints (1 or 4 days), we allow any target TF with increased expression from the previous timepoint to be a regulator TF.

In this manner, we assembled putative regulatory networks throughout the initial stages of differentiation. Over the 3 timepoints, the TF networks grow both in nodes and the number of regulatory interactions (Supplementary Data 2). When examining the nature of the interaction (activation or repression), we find a greater number of activating interactions, suggesting that neuronal differentiation is driven more by activation than by repression of downstream TFs (Fig. 1f). In total, we identified 48 and 403 putative regulator and target TFs, respectively, and 3291 and 1211 unique activation and repression interactions, respectively, between these TFs (Fig. 1g). The rapid growth in the number of regulator and target TFs over the first few days of differentiation suggests a hierarchical model with early TFs more densely interconnected. We find that this is indeed the case: At Day 4, the mean out-degree of early target TFs is ~1.5-fold greater than the mean out-degree of later target TFs (two-sample *t*-test, *p* = 0.01, *n* = 48 TFs at 12 hours and 157 TFs at 24 hours). However, given the observational nature of gene expression and target site chromatin accessibility, it is difficult to know if this greater degree of connection among TFs reflects greater essentiality without direct perturbation of the nodes in the network.

### A massively-parallel CRISPR screen to find essential TFs for neuronal differentiation

Our approach to mapping regulatory interactions takes advantage of concordant changes in genome accessibility and transcript expression to identify downstream TFs involved in *NEUROG1/2* iN differentiation. However, we know little about the importance of each TF (and its downstream targets) on iN differentiation. To understand which downstream TFs are required for iN differentiation, we performed a massively-parallel loss-of-function screen targeting all human TFs. In total, we targeted 1891 TFs with 10 distinct guide RNAs per TF. Guide RNAs in our custom library were optimized for on-target activity and off-target avoidance. We also included 1000 non-targeting guide RNAs to serve as negative control. After cloning the pooled human TF (hTF) library, we verified that the library had a uniform distribution of guide RNAs with minimal bias (5.7-fold difference between 90th and 10th percentile guide RNAs).

**Fig. 2:**
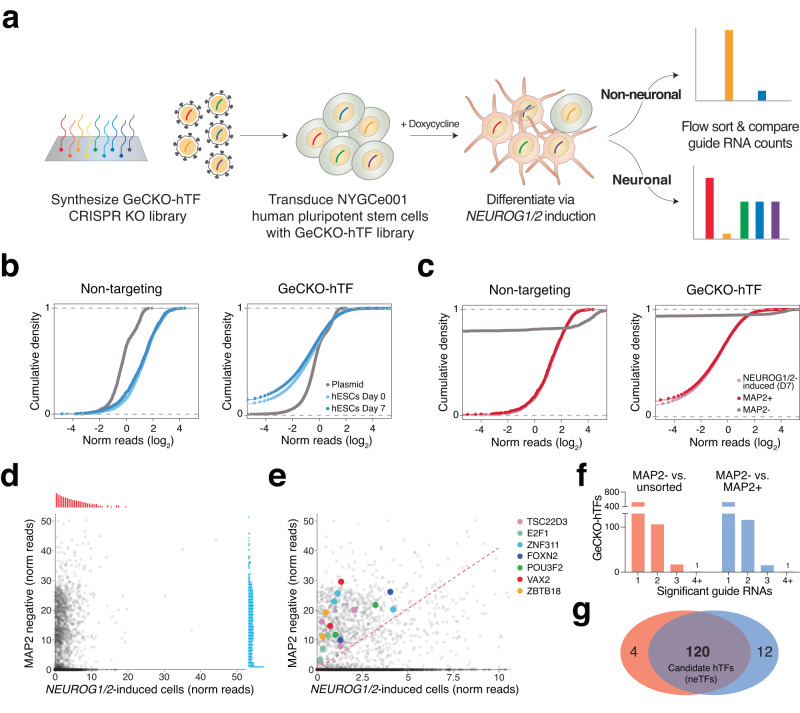
A TFome-scale CRISPR screen in pluripotent cells to identify required TFs for *NEUROG1/2*-induced neuronal differentiation. **a** Schematic of CRISPR screen targeting 1891 hTFs with 10 guide RNAs each. **b** Guide RNA representation for the cloned library and post-transduction library in human embryonic stem cells. Distributions for both non-targeting (negative) control guide RNAs (*left*) and TF-targeting guide RNAs (*right*) are shown. **c** Guide RNA representation for the *NEUROG1/2*-induced cells and MAP2-tdTomato sorted populations. Distributions for both non-targeting (negative) control guide RNAs (*left*) and TF-targeting guide RNAs (*right*) are shown. **d**, **e** Enrichment of guide RNAs in MAP2-negative cells versus all *NEUROG1/2*-induced cells (unsorted). Panel **e** shows an enlargement of enriched guide RNAs from panel **d**. Select TFs with multiple enriched guide RNAs are indicated with colored dots. Red dashed line indicates enrichment of the top 10% of non-targeting guide RNAs. **f** Number of TFs with enriched guide RNAs in the MAP2-negative population compared to either the unsorted population or the MAP2-positive population. **g** Overlap of TFs with multiple enriched guide RNAs (2 or more) in the MAP2-negative population when compared to either the unsorted population or the MAP2-positive population.

To identify essential TFs for iN differentiation, we transduced the hTF library into NYGCe001-A human embryonic stem cells, which carry a knock-in tdTomato fluorescent reporter that is precisely placed in-frame with one allele of MAP2 with a ribosomal skipping 2 A peptide between MAP2 and tdTomato (Fig. 2a)^24^. We confirmed that lentivirally-delivered Cas9 results in high gene knockout efficacy in NYGCe001-A (Supplementary Fig. 2a, b). After hTF library transduction and puromycin selection for 1 week, we added doxycycline to induce *NEUROG1/2* iN differentiation. After 1 week of differentiation, we found that 84% of hTF library transduced cells were tdTomato-positive, indicating that the majority of cells transduced with the hTF library differentiated into iNs (Supplementary Fig. 2c). Untransduced differentiated control cells yielded a similar number of tdTomato-positive cells (87%).

Using fluorescence-activated cell sorting (FACS), we isolated tdTomato-negative cells by setting a gate using isogenic iNs (HUES66) without a MAP2-tdTomato knock-in cassette. We also isolated tdTomato-positive cells by setting another gate using untransduced NYGCe001-A iNs. To keep full library representation, we sorted 10 M cells (500-fold representation of the hTF CRISPR library). Given the long sorting period, we verified after FACS that there was a minimal shift in the percentage of tdTomato-expressing cells in the input population (Supplementary Fig. 2c). In parallel, we also cultured cells without any added doxycycline to measure guide RNA dropout due to targeting of essential hTFs.

We isolated genomic DNA from these samples and then amplified and sequenced the guide RNAs in each population. As expected, representation for the nontargeting (negative control) guide RNAs remained consistent during the culturing of pluripotent stem cells, whereas the representation of targeting TFs decreased. (Fig. 2b). When we examine the depleted guide RNAs, we found that guide RNAs targeting essential TFs were the most highly depleted (Supplementary Fig. 3a, Supplementary Data 3). The most-enriched guide RNAs in the stem cells were those targeting *TP53*, whose loss provides a well-established growth advantage in human pluripotent stem cells^29^.

In the differentiated iN populations, we found virtually no shift in the overall library representation between the input population and the tdTomato-positive sorted population (Fig. 2c). In contrast, we observed a major shift in guide RNA representation in the tdTomato-negative population, consistent with the observation that most library-transduced cells were tdTomato-positive at 7 days post *NEUROG1/2*-induction. We found that ~90% of guide RNAs were lowly represented or absent in the tdTomato-negative sorted population when compared with the FACS input (Fig. 2d), reflecting our stringent tdTomato-negative gate. When examining the guide RNAs present in the tdTomato-negative population, we identified a handful of TFs with multiple guide RNAs each (Fig. 2e). We found a high correlation at the guide RNA level and a similar number of TFs with multiple enriched guide RNAs when comparing tdTomato-negative cells to either the tdTomato-positive population or the FACS input (120 shared TFs, 94% overlap) (Supplementary Fig. 3b, Fig. 2f, g). We termed these 120 TFs as neuron-essential TFs (neTFs), given the enrichment of multiple guide RNAs targeting them in the tdTomato-negative population. These 120 neTFs also have high overlap (81%) with significantly enriched genes in the tdTomato-negative population computed using the well-established RNAi Gene Enrichment Score (RIGER) rank using the weighted-sum method^30^. We selected these 120 neTFs as candidate TFs for further analysis.

### Neuron-essential TFs and their targets during *NEUROG1/2* differentiation

**Fig. 3:**
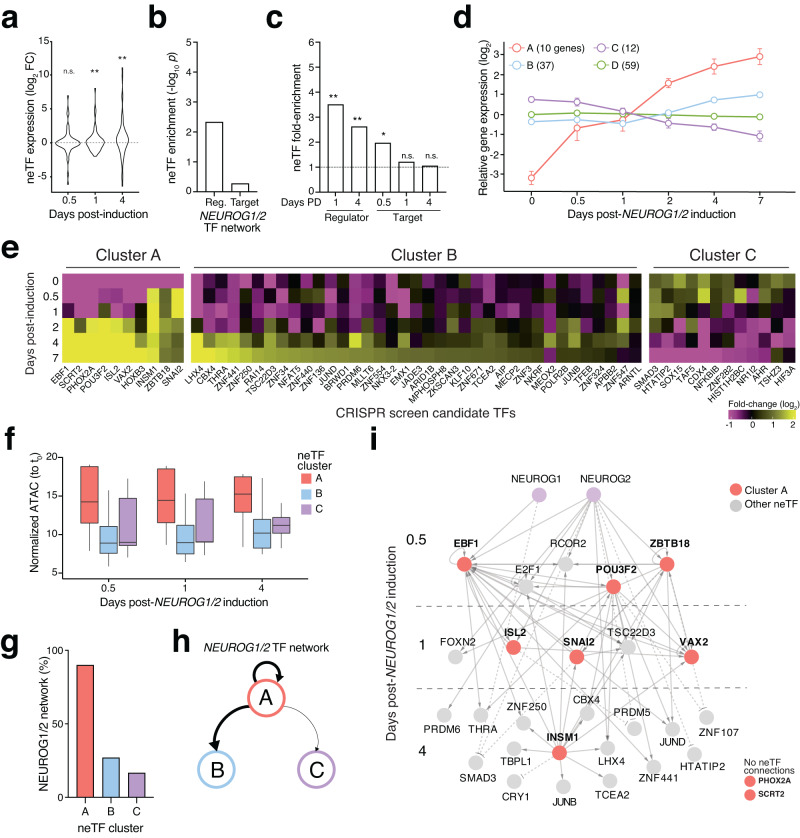
A subset of neuron-essential TFs are characterized by a rapid increase in expression that is accompanied by increased target site accessibility genome-wide. **a** Expression of the 120 neuron-essential TFs (neTFs) after *NEUROG1/2* induction as compared to pre-induction stem cells (*n* = 4 biological replicate inductions). ** indicates *p* < 0.01 (two-sided *t*-test) and *n.s*. indicates not significant (*p* > 0.05). **b** Enrichment *p*-value for neTFs in the TF networks derived from integration of ATAC-seq and RNA-seq datasets in NEUROG1/2-induced cells (*n* = 10^4^ random size-matched sets of TFs). **c** Fold-enrichment for neTFs in regulator-TF or target-TF sets from Fig. 1d. Days PD denotes days post-differentiation (induction). **d** *k*-means clustering of neTF gene expression profiles (*k* = 4). **e** Gene expression fold-change (log_2_) of TFs over different timepoints post-*NEUROG1/2* induction from clusters A, B and C (median of *n* = 4 biological replicates). **f** Normalized open chromatin at the target sites of neTFs from the indicated clusters over time. Boxes show 25th–75th percentiles with a line at the mean; whiskers extend to maximum and minimum values. **g** Quantification of the percentage of cluster A, B or C neTFs in the TF interaction networks shown in Fig. 1d. **h** The regulatory relationship for cluster A, B and C neTFs. **i** Cluster A neTFs in the TF interaction networks shown in Fig. 1d and all other neTFs with direct regulator/target connects to a cluster A neTF.

We first sought to better characterize neTFs by mapping their expression and putative target site accessibility throughout iN differentiation. As a group, we found that neTFs tended to increase in expression during iN differentiation (Fig. 3a), which is expected given that the loss-of-function screen should select for those TFs whose expression is crucial for proper iN differentiation. In addition, neTFs are, on average, lowly expressed or not expressed prior to *NEUROG1/2* induction (Supplementary Fig. 4a). Using our previously constructed TF networks (Fig. 1d), we found that neTFs were enriched in putative regulator TFs that bind *cis*-regulatory elements near target TFs (Fig. 3b). To determine enrichment, we computed empirical *p*-values by repeatedly resampling a size-matched set of TFs (120 TFs). We also performed this analysis at different stages of iN differentiation and found that neTFs were enriched for putative regulator TFs at both early (Day 1) and late (Day 4) stages of differentiation (Fig. 3c). In contrast, neTFs were only enriched for target TFs (that is, targets of regulator TFs) at the earliest timepoint (12 hours).

Given this enrichment in putative regulator TFs at early and late stages of differentiation, we wondered whether we might be able to further dissect different categories of neTFs that are responsible for different stages of neuron differentiation. Towards that end, we performed unsupervised clustering on the expression profiles of neTFs through differentiation (Supplementary Data 4). We found neTF subgroups that begin to be expressed early in differentiation (cluster A) and others expressed later in differentiation (cluster B) (Fig. 3d, e). Cluster A neTFs increased in expression rapidly within 12 to 24 hours of NEUROG1/2 induction, whereas neTFs in cluster B tend to increase at day 2 or 4 after NEUROG1/2 induction. Given that the majority of neTFs have increased gene expression (Fig. 3a), we also identified a small subset of neTFs (cluster C) that initially increase in expression (through 24 hours) and then later decrease in expression during differentiation (Day 2 though 7). These neTFs may regulate target genes whose expression requires precise temporal control. We also identified a subset of neTFs (cluster D) with only minor changes in expression during differentiation and did not include these in our subsequent analyses.

Using our TF interaction networks (Fig. 1d), we found that the targets of cluster A neTFs harbor ~2-fold greater open chromatin at the neTF binding sites than cluster B or C neTFs (Fig. 3f). Thus, the rapid increase in the expression of cluster A neTFs is accompanied by greater chromatin accessibility than for other neTFs. Given their rapid increase in expression and greater target site accessibility, we hypothesized that cluster A would be enriched for regulatory TFs that connect to downstream target TFs. Using a similar randomization approach as before, we found that indeed cluster A neTFs are enriched in putative regulator TFs, emphasizing their key role in driving iN differentiation (Supplementary Fig. 4b).

Among the neTFs, we found that nearly all cluster A neTFs were present in the TF interaction network (9 out of 10 cluster A neTFs), whereas a smaller fraction of cluster B and C neTFs were present (Fig. 3g). To understand if the inclusion criteria (TF expression and putative binding site accessibility) might be responsible for these differences among neTFs, we substantially loosened RNA-seq and ATAC-seq significance thresholds and re-built the interaction networks. As expected, this led to a large increase in the number of inferred TF interactions (5517 to 8658 TF interactions). However, even in these new interaction networks, we found that the majority of cluster B and C neTFs were not present as either regulators or targets (Supplementary Fig. 4c). Thus, the Neurogenin TF network assembled from multi-omic phenotyping over differentiation accurately capture major regulators (cluster A) but misses many essential TFs for differentiation identified in the knockout screen.

For those cluster B and C neTFs that were present in the TF interaction network, we found a striking hierarchical relationship with cluster A neTFs: Specifically, cluster A neTFs were always regulators of cluster B or C neTFs but never vice-versa (Fig. 3h), as can be seen by examining the subnetwork containing cluster A neTFs and all first-order (direct) connections to other neTFs (Fig. 3i and Supplementary Fig. 4d). Thus, within the neTFs, those from cluster A tend to be closely linked to other neTFs (and *NEUROG2* or *NEUROG1*) and, given their early activation, sit atop a hierarchy of control over neTFs from other groups.

### Top-ranked neuron-essential TFs disrupt neurogenesis and are conserved in mouse neuron differentiation

**Fig. 4:**
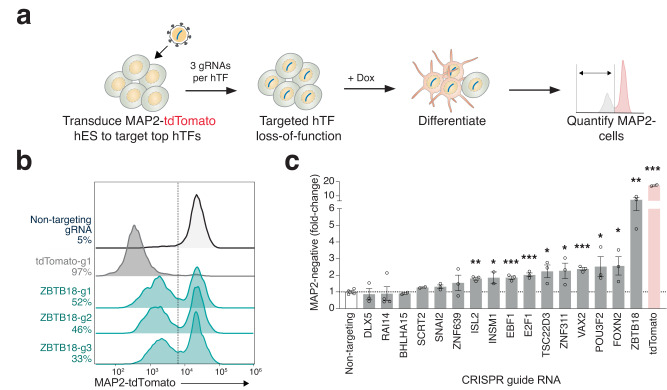
Loss of top-ranked neuron-essential TFs disrupts neurogenesis. **a** Targeting of individual neuron-essential TFs in human embryonic stem cells to quantify MAP2-negative cell numbers. **b** Flow cytometry of MAP2-tdTomato expression at 7 days after *NEUROG1/2*-induction in cells transduced with Cas9 and guide RNAs targeting ZBTB18 (3 distinct guide RNAs), tdTomato (1 representative guide RNA), or a non-targeting (negative) control (1 representative guide RNA). **c** Change in MAP2-negative cells (compared to non-targeting control) when targeting the indicated gene (*n* = 3 guide RNAs per gene).

We next sought to examine a subset of neTFs individually in order to understand whether loss of each TF prevents iN differentiation (Fig. 4a). To that end, we cloned 3 individual guide RNAs for 16 different neTFs and transduced them into NYGCe001-A pluripotent stem cells. Of these 16 TFs, we selected half of them from cluster A neTFs (EBF1, SCRT2, POU3F2 [BRN2], ISL2, VAX2, INSM1, ZBTB18, and SNAI2). We also cloned positive control (tdTomato-targeting) and negative control (nontargeting) guide RNAs. As in our pooled screen, we selected transduced NYGCe001-A pluripotent stem cells for 7 days before adding doxycycline to trigger iN differentiation. At day 7 post-doxycycline addition, we quantified the percentage of tdTomato-negative cells by flow cytometry (Fig. 4b). Nearly all of the targeted TFs had an increase in tdTomato-negative cells relative to the negative control guide RNAs (Fig. 4c). Interestingly, the top four TFs whose loss led to the largest increase in tdTomato-negative cells (VAX2, POU3F2, FOXN2 and ZBTB18) were all predicted as direct targets of *NEUROG2* in the TF networks derived from expression and chromatin accessibility datasets (Fig. 1d). Of the targeted TFs, we noticed the greatest increase in tdTomato-negative cells for ZBTB18-targeting guide RNAs. In addition, we found that ZBTB18, POU3F2 (BRN2) and VAX2 could potentially activate the expression of other neTFs, suggesting a hierarchical relationship among the neTFs (Supplementary Fig. 4d). For these neTFs, we also see a substantial increase in open chromatin at their promoters over the course of iN differentiation (Supplementary Fig. 5).

To further validate top-ranked neTFs and understand whether these neTFs are broadly conserved, we conducted a similar TFome-wide CRISPR screen using NEUROG2-induced mouse embryonic stem cells. To select for neuronal cells, we developed a *TUBB3*-GFP knock-in line at the endogenous *TUBB3* locus in A17 mouse embryonic stem cells^15^ (Supplementary Fig. 6a). In contrast to the human *NEUROG1/2*-induced differentiation screen, we observed less dropout of guide RNAs, indicating that the selection (for TUBB3-GFP negative cells) was less strict than in the human TF screen (Supplementary Fig. 6b, c, Supplementary Data 5). Despite this difference, we found a remarkable degree of overlap between enriched TFs in the GFP-negative population and the human neTFs using the same enrichment criteria. Out of the 120 neTFs from the human screen, we identified 45 overlapping neTFs in the mouse neuron differentiation screen (Supplementary Fig. 6d). Notably, 6 of the 10 cluster A neTFs (SCRT2, POU3F2, HOXB3, INSM1, SNAI2 and ZBTB18) identified from the human screen were also top-ranked TFs in the mouse screen. Despite substantial differences in the two TFome-wide CRISPR screens (organisms, neural differentiation methods, and reporter genes), the ability to detect many of the same neTFs suggests these TFs are conserved bona fide essential TFs for neuron differentiation.

### ZBTB18 is a master regulator of other neTFs and its loss produces iNs with stunted dendritic arborization

ZBTB18 (also known as ZNF238, ZFP238 and RP58) is expressed in neuronal progenitors and postmitotic neurons in different brain regions including the developing mammalian cerebral cortex and cerebellum where it is required for neuronal differentiation (Xiang et al.^26^). It functions as a transcriptional repressor most likely via its association with DNA methytransferases and histone regulators (Xiang et al.^26^; Fuks et al.^31^; Xiang et al.^27^). Using human fetal brain tissue (Allen BrainSpan, *n* = 20 donors), we observed that ZBTB18 reaches peak expression at the end of the first trimester (Supplementary Fig. 7a). This corresponds to the same developmental period where we previously found maximal correlation of gene expression between iNs and human fetal cortex (Supplementary Fig. 1c).

**Fig. 5:**
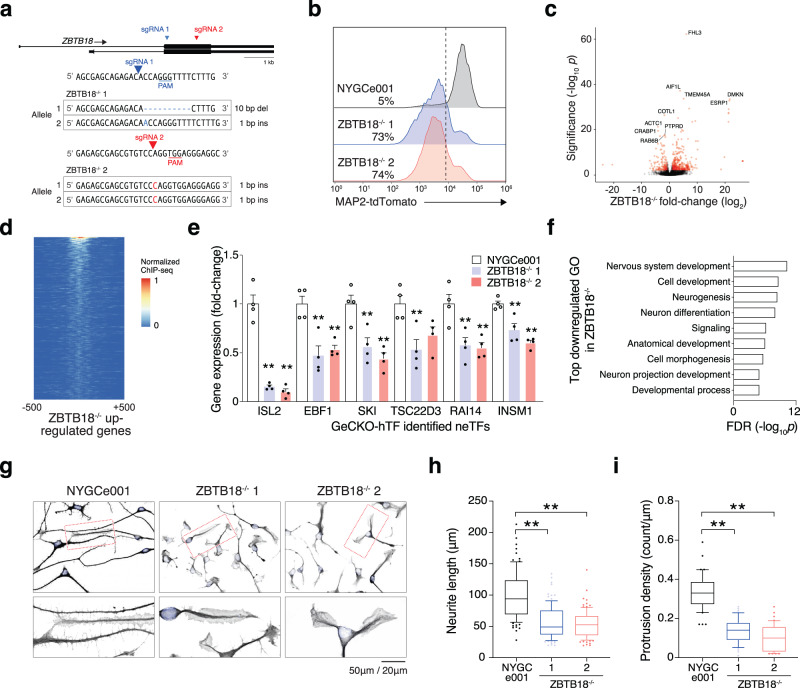
*ZBTB18*-null cells have widespread alterations in gene expression and result in immature neurons with stunted neurite development. **a** Schematic illustration of the strategy for establishing ZBTB18-null hESCs using CRISPR/Cas9 genome editing with 2 different guide RNAs. **b** Flow cytometry of MAP2-tdTomato from induced neurons (iNs) at day 7 after *NEUROG1/2*-induction from NYGCe001 (parental) and ZBTB18^-/-^−1 and ZBTB18^-/-^−2 (isogenic knockouts). **c** Volcano plots for differential gene expression of ZBTB18^-/-^ iNs at day 4 after *NEUROG1/2*-induction (*n* = 4 replicates per genotype and timepoint). Red dots indicate genes with *p*_*adj*_ < 0.01 and |fold-change | > 2. **d** ZBTB18 binding near upregulated genes via chromatin immunoprecipitation with sequencing in HEK293T cells (*n* = 2 biological replicates, data is from ref. ^32^). **e** Other neuron-essential TFs with significant changes in expression in ZBTB18^-/-^−1 and ZBTB18^-/-^−2 at day 4 after *NEUROG1/2*-induction. **f** Top-ranked Gene Ontology categories in significantly altered genes in ZBTB18-null iNs at day 4 after *NEUROG1/2*-induction. **g** Representative epifluorescence images of WT, ZBTB18^-/-^−1 and ZBTB18^-/-^−2 iNs at day 4 after *NEUROG1/2*-induction. **h**–**i** Quantification analysis for neurite length **h** and protrusion density **i** for WT, ZBTB18^-/-^−1 and ZBTB18^-/-^−2 iNs at day 4 after *NEUROG1/2*-induction.

Of the neTFs that we knocked-out individually, we found that loss of ZBTB18 triggered the greatest loss of MAP2+ cells, several fold higher than any other neTF. To characterize the mechanisms underlying this large impact on differentiation, we engineered two biallelic ZBTB18 knockout lines via transient transfection of Cas9 with two different guide RNAs (Fig. 5a). After the introduction of the guide RNAs, we isolated single-cell clones and confirmed ZBTB18 loss via Sanger sequencing of both alleles (Supplementary Fig. 7b). As expected, with both guide RNAs, we found that insertions/deletions occurred at cut sites of the respective guide RNAs. In one ZBTB18-null cell line, we identified a 10 nt deletion on one allele and a 1 nt insertion (adenosine) on the other allele. For the second ZBTB18-null cell line, both alleles had a 1 nt insertion (cytosine). In agreement, differentiated cells from the ZBTB18-null cell lines did not express ZBTB18 as compared to the isogenic parental cells as detected using a previously characterized ZBTB18 antibody^27,32^ (Supplementary Fig. 7c). After *NEUROG1/2* induction, both ZBTB18-null cell lines did not differentiate efficiently as measured by flow cytometry of the MAP2-2A-tdTomato reporter (Fig. 5b). Compared to the parental cell line, we found that both ZBTB18-null cell lines had ~15-fold more tdTomato-negative cells—an even stronger phenotype than the polyclonal population of ZBTB18-targeted cells (Fig. 4c).

To determine if ZBTB18 loss triggers widespread aberrant gene expression, including de-repression of silenced genes, we performed RNA-sequencing on differentiated cells from both ZBTB18-null cell lines and identified 1034 genes with significant changes in expression compared to the parental cell line (Fig. 5c). As expected, we identified a greater number of genes with increased gene expression (de-repression) upon ZBTB18 loss (741 genes) than genes with reduced expression (293 genes). In contrast, undifferentiated ZBTB18-null cell lines had relatively few differentially-expressed genes compared to the isogenic controls (Supplementary Fig. 8a–c), indicating that these large changes in gene expression required differentiation.

To determine if dysregulated genes are bound by ZBTB18, we used a published ZBTB18 chromatin immunoprecipitation (ChIP-seq) dataset from the ENCODE Consortium to associate TF binding to the gene expression changes that we observed. Despite the different cell types, we found that genes with increased expression contained ZBTB18 ChIP peaks in their *cis*-regulatory regions^33^ (Fig. 5d). Compared to genes with decreased expression, there was a 3-fold enrichment in ZBTB18 binding sites in upregulated genes even at more strict significance thresholds. (Supplementary Fig. 8d). Several of the neTFs that we identified in our CRISPR screen were also reduced in expression upon ZBTB18 loss. (Fig. 5e). We hypothesized that ZBTB18 loss might result in more severe impairments in neuron differentiation due to loss of both target gene repression and indirect effects mediated via reduced expression of other neTFs (and their targets). Of the 120 neTFs from the CRISPR screen, we found that 52 neTFs harbor ZBTB18 ChIP peaks proximal to these genes.

The differentially expressed genes were highly enriched not only for genes involved in nervous system development but also for genes involved in neuron projections and cell morphogenesis (Fig. 5f, Supplementary Fig. 8d), which suggested that there may be further defects in the cytoskeleton and neurite formation. To characterize the cytoskeleton and neurite projections, we immunostained differentiated cells from all cell lines for β3-tubulin and found that neurites from differentiated ZBTB18-null cells were markedly different than those from the parental cell line (Fig. 5g). We observed short, stunted neurites with few protrusions or spines (Fig. 5h, i). On average, neurite length was reduced by ~50% and protrusion density was reduced by nearly 70% (two-sample *t*-test, *p* < 0.01). Taken together, our data showed that ZBTB18 loss alters the expression of many genes, including several neTFs, and results in defective neuronal differentiation and maturation that includes stunted development of neurites and dendritic spines in differentiated cells.

## Discussion

Neurogenins were first identified as proneural genes over two decades ago^2,3,6,34^. More recently, the expression of *NEUROG2* alone or in combination with *NEUROG1* has recently been shown to convert pluripotent stem cells into spiking neurons (Busskamp et al.^16^; Lu et al.^17^; Zhang et al.^18^). However, our understanding of the mechanisms—and specifically downstream TFs—driving Neurogenin-directed neuronal differentiation has been limited. In this study, we identified several neuron-essential TFs where gene loss-of-function significantly hindered *NEUROG1/2* directed neuronal differentiation. We found that these TFs form a hierarchical gene regulatory network and act in distinct cascades to promote neuronal differentiation.

### Integrative functional genomics to identify neuron essential TFs for directed differentiation

To pinpoint downstream TFs required for *NEUROG1/2* directed neuronal differentiation, we developed an integrative approach that combines TF gene expression, chromatin accessibility and TF knock-out. We first built putative TF networks activated by *NEUROG1/2* through open chromatin profiling of *cis*-regulatory elements (CRE) near TF genes, TF binding site localization within these CREs, and TF gene expression during iN differentiation. From this, we identified ~900 TFs with significant changes in gene expression, suggesting many TFs might contribute to *NEUROG1/2* directed neuronal differentiation. To understand which of these were truly *essential* for neuronal differentiation, we developed a pooled Cas9 knockout screen with a CRISPR library that targets all human TFs with 10 independent CRISPR constructs per TF. In this manner, we were able to identify TFs whose loss inhibits neuronal differentiation as reflected by a fluorescent reporter of MAP2 expression. From this TFome-wide CRISPR screen, we identified 120 neuron-essential TFs (neTFs) as candidates for further characterization.

In contrast to previous work searching for downstream effectors of NEUROG2 via cDNA subtractive hybridization^22^, microarray profiling^35^, or in silico mutation analysis^9^, our CRISPR screen directly connects loss of specific TFs to defects in differentiation. The subtractive hybridization screen identified only 10 TFs with differential expression in *NEUROG2*^*+/-*^ mouse telencephalon—likely limited by the inherent lower sensitivity of subtractive hybridization cloning methods^22^. Later work using *NEUROG2* overexpression in chick embryos found over 1000 early response genes (including *ZBTB18*)^35^. Our study presents a different approach to prior methods that quantify gene expression after *NEUROG2* ablation or overexpression by instead perturbing downstream target TFs.

After evaluating these candidate neTFs in the developing iNs, we found a subgroup (Cluster A) with a rapid increase in expression and greater chromatin accessibility at their target sites in response to *NEUROG1/2* induction. Using the TF interaction networks, we found that half of the neTFs from Cluster A were predicted direct targets of *NEUROG1* or *NEUROG2* and that nearly all of them were contained within the larger *NEUROG1/2* regulatory network. In addition, TFs from Cluster A were more likely to be serve as regulators of other TFs in the network and of other neTFs. Subsequent knock-out of candidate TFs confirmed that those in Cluster A had the reduction in MAP2+ neuron differentiation, suggesting that the integrative approach combining expression, genome accessibility and loss-of-function screens can better identify key TFs. Of course, further studies at longer timepoints will be needed to distinguish whether those key TFs are required for differentiation or simply delay differentiation. This integrative approach could also aid in the study of other TF regulatory networks involved in the differentiation of various cell types. Although several recent studies examine such regulatory networks by bringing together gene expression and chromatin accessibility, such as recent work in CD4 + T cells or epidermal differentiation^36,37^, few integrate such multi-omic datasets with massively-parallel mutagenesis or loss-of-function^38,39^.

### Similarities between directed neuron differentiation models and in vivo neural differentiation

In vivo expression of NEUROG1 and NEUROG2 triggers a genetically programmed cascade of TFs along with other target genes^4,40,41^. They play a key role in specifying neuronal over glial fates^42,43^, dorsal over ventral patterning^40^ and patterning of dendritic morphologies characteristic of pyramidal neurons^44^. Previous reports have shown that ectopic expression of Neurogenins in *Xenopus* or zebrafish promote the generation of neural progenitors and promote neuronal differentiation through activation of TF from *ISL*, *EBF* and *LHX/LIM* families^34,45–47^. Interestingly, in the iN model, we also found a rapid up-regulation of human TFs from these families after Neurogenin induction with several members identified as neuron essential in our CRISPR screen. In particular, several cluster A neTFs (PHOX2A, POU3F2 [BRN2], EBF1, VAX2, INSM1, SCRT2) were previously identified as key TFs for early neural development in model organisms^48–52^. Many downstream target TFs of Neurogenins may be shared between directed human neuron differentiation and in vivo neuralization in diverse model organisms, suggesting that these TF interaction networks are largely conserved across species, although this observation will require careful validation in future comparative genomic studies.

### ZBTB18 plays a central role in the hierarchical TF network activated by Neurogenins

ZBTB18 is a zinc finger containing TF that plays a major role in brain development and in vivo neuronal differentiation^53,54^. Although we identified several essential TFs for *NEUROG1/2* directed neuronal differentiation, loss of ZBTB18 produced the most dramatic defects in neuronal differentiation upon validation with single TF perturbations. Previous studies have shown that ZBTB18 is required for the maturation and survival of the excitatory neurons of the cerebral cortex^55^. Germline mutations in ZBTB18 in humans result in abnormal brain development, such as microcephaly and intellectual disability, and late-acquired somatic mutations can trigger certain brain-associated cancers^27,56,57^. We also identified ZBTB18 as an essential TF for *NEUROG2*-directed neuronal differentiation from the mouse CRISPR screen. To help understand the mechanisms responsible for the striking effects of ZBTB18 loss on directed in vitro neuronal differentiation, we further characterized its role using knockout lines. We confirmed that biallelic ZBTB18 loss resulted in major alterations in neuron morphology: short neurites with minimal protrusions. Loss of ZBTB18 also triggered down-regulation of several other neTFs identified from the screen, including EBF1 and TSC22D3^58,59^. EBF1 and TSC22D3 were also predicted ZBTB18 targets in the TF interaction network constructed from our expression and chromatin accessibility datasets. Within the hierarchy of TF activation, our data indicates that ZBTB18 occupies a position near the apex of the TF network and acts to regulate the expression of several other neTFs. Future work is still needed to better understand the hierarchical relationships between ZBTB18 and other neuron-essential TFs.

With new methods to uniting high-throughput perturbations with single-cell, multi-omic readouts and organoid models capable of producing diverse human cortical neurons^60–63^, it may soon be possible to go beyond loss-of-function screens for neuralization (e.g. MAP2 and Tubb3 fluorescent reporters) and understand the exact TF cascades responsible for multiple cortical neuron subtypes.

## Methods

### Cell culture and differentiation

All human stem cell experiments were conducted following prior approval from the New York Genome Center Institutional Stem Cell Research Oversight (ISCRO) committee and Institutional Biosafety Committee. NYGCe001-A human embryonic stem cells were derived from HUES66 (Harvard Stem Cell Institute, NIH hESC-10-0057, 46XX/female) and contain doxycycline-inducible *NEUROG2-*2A*-NEUROG1-*2A-PuroR (via lentiviral integration) and a 2A-tdTomato knock-in at the *MAP2* locus, triggering expression of a red fluorescent protein upon neuronal differentiation via doxycycline induction^24^. Stem cells were maintained using a slight modification of the Enhanced Culture Platform as described previously^64^. Briefly, NYGCe001-A cells were cultured in Essential 8 media (Thermo) supplemented with 100 μg/mL Normocin (InvivoGen) and cultured in standard tissue culture dishes coated with Geltrex (Thermo) at 37 °C in 5% CO2. Accutase (STEMCELL) was used for passaging the cells. 10 μM Rho kinase inhibitor (ROCKi) Y-27632 (MilliporeSigma) was added to the culture medium at each passage. ROCKi was removed at the subsequent media change (typically 24 hours later).

To generate *NEUROG1/2* induced neurons (iNs), on day 0, NYGCe001-A cells were plated in the presence of 1 μg/ml doxycycline (Sigma) in Essential 8 medium. The cells were plated in Geltrex-coated 15 cm cell culture dish at a density of 3.5 million per dish for fluorescent activated sorting. The cells were selected in puromycin (1 μg/ml) from day 1 to day 4 in Essential 8 medium with gradually increasing Neurobasal (NB) medium (Essential 8 to NB ratio: 3:1 on day 1, 1:1 on day 2, and 1:3 on day 4). NB medium was supplemented with 2% B27 and 1x penicillin-streptomycin (all from Thermo). On day 4, the puromycin was removed, and the culture medium was changed to 100% NB. Doxycycline (1 μg/ml) was present throughout the entire culture period after *NEUROG1/2* induction.

### Human and mouse TFome CRISPR knock-out libraries

For the human/mouse TF library, we designed guide RNAs to target 1891 known human TFs or 1682 known mouse TFs using the GUIDES web tool (http://guides.sanjanalab.org) with up to 10 guide RNAs per TF and also included 1000 non-targeting (negative control) sgRNAs^65^. Synthesized oligonucleotides (Twist Bioscience) were dissolved in Buffer EB (Qiagen). 16 ng/ul single stranded pooled oligos were amplified with NEBNext High-Fidelity 2x PCR Master Mix (NEB) with following PCR protocol: 98 °C for 30 s, 8 x [98 °C for 10 s, 63 °C for 10 s, 72 °C for 15 s], 72 °C for 3 min. Amplified oligos were cloned into the lentiCRISPRv2-FE-Zeo vector. We cloned lentiCRISPRv2-FE-Zeo (all-in-one vector with Cas9 and a FE-modified guide RNA scaffold and Zeocin resistance) from our prior lentiCRISPRv2 (Addgene #52961)^66^ vector. For oligo cloning, we first digested 40 ug of vector with Esp3l (Thermo) in 10x FastDigest Buffer (Thermo) and 1 mM DTT in 100 uL for 30 minutes at 37 °C. After 30 minutes, we added 10 ul of FastAP Thermosensitive Alkaline Phosphatase (Thermo) and 10x FastDigest Buffer in a 200 µL total volume. Gel-purified amplified were cloned into cut lentiCRISPRv2-FE-Zeo vector using 2x Gibson Assembly Master Mix (NEB) with 10 times molar ratio of pooled oligos to digested vector and transformed to electrocompetent Endura bacterial cells (Lucigen) and plated on LB+Amp plates. We recovered ~560 colonies per construct and then purified the library using 4 Plasmid Plus Maxiprep (Qiagen) columns.

### Lentiviral production and transduction

To produce lentivirus, 120 million 293FT cells (Thermo) were plated on eight T-225 flasks the day before transfection in D10 media: DMEM (Caisson Labs) supplemented with 10% Serum Plus II Medium Supplement (Sigma-Aldrich). One hour prior to transfection, media was changed with 15 ml of fresh, pre-warmed D10 per flask. For each flask, 15 μg human TF GeCKO (hTF-GeCKO) transfer plasmid, 8.25 μg pMD2.G, 12 μg psPAX2, 1.5 mL OptiMEM (ThermoFisher, 31985070) and 82.5 μg of 1 mg/ml PEI-Max (Polysciences) were mixed together and then added to cells. At 4 hours after transfection, the media was changed to pre-warmed D10 supplemented with 1% Bovine Serum Albumin (Thermo). At 60 hours after transfection, the media was removed and centrifuged at 3000 rpm in an Allegra X-30R (Beckman) at 4 °C for 5 min to pellet cell debris. The supernatant was filtered through 45 μm PVDF filters (CellTreat). The supernatant was then ultracentrifuged for 2 hours at 100,000 g in a Sorvall Lynx 6000 and the pellet was resuspended overnight at 4 °C in PBS. After viral titration, we transduced 80 million NYGCe001-A embryonic stem cells with hTF-GeCKO library lentivirus to achieve a MOI of ~0.3. At 2 days after transduction, we added 4 μg/µL Zeocin (Thermo) to the media. Cells were maintained in selection media for 2 days with ROCKi and then returned to standard (Essential 8) media. After selection, cells were passaged whenever they reached approximately 75% confluence. After each passage, at least 10 million cells were maintained in culture to ensure >500-fold coverage of the total number of guide RNAs in the hTF-GeCKO library.

### Flow cytometry and sorting

The cells were dissociated into a single cell suspension using Accutase and passed through a 35 μm cell strainer (BD Falcon). Flow cytometry data was acquired on BD FACSAria II for the CRISPR human and mouse CRISPR screen sorting or a Sony SH800 for the validation experiments and analyzed using FlowJo v10.5 (BD).

### Genomic DNA isolation, guide RNA amplification and quantification

We used a two-step PCR protocol (PCR1 and PCR2) to amplify the guide RNA cassette for Illumina sequencing^66^. Briefly, genomic DNA (gDNA) was extracted using a previously described protocol^67^. For the first PCR reaction, we amplified 10 μg gDNA for each sample with Taq-B polymerase in 100ul PCR reaction (Enzymatics). 5ul PCR1 products were then used for amplification with barcoded PCR2 primers with Q5 polymerase (NEB). PCR2 products from each sample were normalized and combined, then gel-purified from a 2% E-gel EX (Life Technologies) using the QiaQuick gel extraction kit (Qiagen). The purified, pooled library was then quantified with Tapestation 4200 (Agilent Technologies). Sequencing was performed on the NextSeq 550 instrument using the HighOutput Mode v2 with 75 nt single-end reads (Illumina). Sequencing reads were demultiplexed upon sequencing based on Illumina i7 barcodes present in PCR2 reverse primers using Illumina BaseSpace. We performed adaptor trimming by treating the hU6 promoter sequence as a 5’ adapter, using cutadapt v1.13 [-e 0.2 -O 5 -m 20 -g TCTTGTGGAAAGGACGAAACACCG]. Processed guide RNA sequences were aligned to the appropriate library reference (hTF or mTF) allowing for up to 1 mismatch using bowtie v1.1.2 [-a --best --strata -v 1 –norc] with alignment rates of 69% to 77%.

### Computational analyses of human and mouse CRISPR screens

To look for neuron-essential human TFs for *NEUROG1/2* induced neuronal differentiation, we analyzed the changes in sgRNA distribution in tdTomato-negative cells compared to either the unsorted input or the tdTomato-positive cells. To look for enriched mouse TFs for *NEUROG2-*induced neuronal differentiation, we analyzed the changes in sgRNA distribution in GFP-negative cells compared to either the unsorted input or the GFP-positive cells. For each comparison, we first assessed how many guide RNAs for each TF were enriched above the top 10th percentile of non-targeting guide RNAs (empirical FDR < 0.1). With these enriched guide RNAs, TFs were sorted based on the number of guide RNAs targeting each TF present. The enriched TFs with 2 or more enriched guide RNAs were selected for comparison. The neuron-essential human TFs were defined as those TFs shared between the two groups (tdTomato-positive compared to either the FACS input or tdTomato-positive). We also used the RNAi Gene Enrichment Ranking (RIGER) method to compute the enrichment score of guide RNAs for each transcription factor^30^. RIGER is more robust to sparse samples (e.g. the tdTomato-negative population) than other methods because it calculates a weighted sum of the top two guides, rather than considering all guides designed for each gene. Specifically, guide RNAs are ranked, and a weighted ranking of the top two most enriched guide RNAs is computed for each gene, representing a ranking score. RIGER uses permutation of gene labels to compute the *p*-value of each RIGER rank and determine significance.

### Immunocytochemistry

Cells were grown on Geltrex-coated coverslips and fixed with 4% paraformaldehyde (Sigma) and 4% sucrose in PBS at room temperature for 5 min followed by washing 3 times with PBS. The fixed cells were then permeabilized with 0.1% Triton X-100 (Sigma) in PBS for 5 min, washed 3 times with PBS, and incubated in 3% bovine serum albumin (Sigma) in PBS for 1 hour to block nonspecific binding. The cells were incubated overnight with the following primary antibodies at 4 °C overnight: guinea pig anti-MAP2 (188004, 1:1000; SYnaptic SYstems) or rabbit anti- β3-Tubulin (302302, 1:5000; SYnaptic SYstems). After the cells were washed 3 times with PBS, they were incubated the secondary goat anti-rabbit, Alexa Fluor 555 (A21428, 1:1000; Thermo) or donkey anti-guinea pig, Alexa 647 (706-605-148, 1:1000; Jackson ImmunoResearch) at room temperature for 1 hour. The images were collected using an Axio Observer Z1 microscope (Zeiss).

### Western blots

Cells were washed with ice-cold PBS twice and then scraped off the plate in ice-cold RIPA Buffer (Cell Signaling) with freshly added Protease Inhibitor Cocktail (Sigma P8340). Cell lysate was transferred to a 1.5 ml microcentrifuge tube and incubated for 30 min at 4 °C with constant agitation. The soluble fraction of the lysate was isolated by centrifugation for 10 minutes at 14,000 x g at 4 °C. The BCA assay was used to quantify protein concentrations (ThermoFisher). For each blot, 35 μg of protein was loaded onto a 4-12% Tris-Glycine precast gel (ThermoFisher) and run at 120 V for 1.5 hour at room temperature. Proteins were transferred onto a nitrocellulose membrane (BioRad) at 100 V for 1 hour at 4 °C in a 20% methanol transfer buffer. Immunoblots were blocked with 5% skim milk dissolved in 1x TBS with 1% Tween-20 (TBST) and incubated overnight at 4 °C with anti-ZBTB18 antibody (generous gift of Nadia Dahmane)^32^. Then, the blot was incubated with IRDye 680RD donkey anti-rabbit (0.2 mg/mL, LI-COR 926-68073). The blot was also incubated with anti-GAPDH antibody (Cell signaling Technology, 97166 S) as a loading control for 1 hour at room temperature, and then with IRDye 800CW donkey anti-mouse (0.2 mg/mL, LI-COR 926-32212). The blots were imaged using Odyssey CLx (LI-COR).

### RNA-sequencing and differential expression analyses

RNA was extracted from NYGCe001-A cells and iNs at multiple timepoints after differentiation using the Quick-RNA Mini kit (Zymo) and prepared using the TM3’seq method^68^. Sequencing was performed using 37 nt paired-end reads on a NextSeq 550 (Illumina). Each sample was prepared with at least four independently differentiated biological replicates.

RNA-seq FASTQ files from biological and technical replicates were trimmed by Cutadapt (v1.13) to remove the 3’ trailing polyA (-a AAAAAAA -m 25 -O 5) sequences. Trimmed FASTQ files were aligned to the hg38 reference genome (GENCODE release 31) using STAR (v2.7.1a). RSEM (v1.2.21) was used to generate count matrices for each replicate. Count matrices (expected_count from RSEM output) were then used for downstream analyses. We used the DESeq2 R package to analyze differential gene expression with RSEM count matrices as input. The low read count threshold was set at 50 total reads across 4 replicates. For different timepoints after *NEUROG1/2* induction in wild-type NYGCe001 cells, contrasts were constructed based on time points after doxycycline induction versus stem cell stage (design=~timepoint). For ZBTB18-null samples, contrasts were constructed based on knockout genotype versus isogenic (parental) cells (design=~genotype+timepoint+genotype:timepoint). Gene Ontology analyses were performed using GOrilla^69^. ZBTB18 ChIP-seq data is from a previously published HEK293 dataset (GSM2026861)^33^.

### ATAC-seq library preparation and peak identification

We performed ATAC-seq as previously described with an optimized lysis buffer to lower mitochondrial contamination^60,70^. Briefly, cell membranes were lysed in Nuclei Buffer (10 mM Tris-HCL pH 7.4, 3 mM MgCl2, 10 mM NaCl) with 1% Tween-20 freshly added. After pipetting up and down, nuclei were isolated by centrifugation at 500 xg for 5 minutes at 4 °C. After discarding the supernatant, nuclei were resuspended in Tagmentation DNA (TD) Buffer with transposase (TnY) and incubated at 37 °C for 30 minutes. We prepare a 2x TD Buffer stock as follows: 20 mM Tris-HCl (pH 7.4) and 10 mM MgCl_2_ in ultra-pure water. After purification on a MinElute column (Qiagen), the purified, tagmented DNA was PCR amplified using Pfu-X7 polymerase and barcoded primers for 10 cycles as follows: 72 °C for 5 min, 98 °C for 30 s, 10 x [98 °C for 10 s, 63 °C for 30 s, 72 °C for 1 min], 4 °C hold. Detailed protocols for purification of these enzymes are available in Liscovitch-Brauer*, Montalbano* et al.^60^. The PCR product was purified via a 1.5X SPRI cleanup (Agencourt) and checked for a characteristic nucleosome banding pattern using a TapeStation (Agilent). Samples were sequenced with paired-end 37 nt reads on a NextSeq 550 (Illumina). All samples were processed with at least 2 biological replicates that were separately cultured, tagmented and analyzed.

ATAC-seq FASTQ files were aligned to the hg38 reference genome (GENCODE release 31) using BWA (v0.7.17) using default pair-end mode parameters (bwa sampe). SAMtools (v1.9) was used to sort, index, and filter read alignments. Properly paired reads with alignment score greater than 20 were kept for downstream analysis. We used MACS2 (v2.1.2) to call peaks on filtered ATAC-seq alignments using the recommended pair-end mode for ATAC-seq alignments (–format BAMPE –gsize hs –qvalue 0.05 –cutoff-analysis --bdg). Peaks that reside in ENCODE Blacklist regions were removed. Filtered peaks were used as inputs for downstream analysis. Consensus peaks, along with count matrices were generated using the DiffBind R package. Consensus peaks were defined as peaks present in at least two samples from any time point. For all ATAC read counts, we computed them using the dba.count function from diffbind with parameters (score=DBA_SCORE_READS minOverlap=2).

### Transcription factor interaction networks

We sought to integrate our expression data (RNA-sequencing) and chromatin accessibility (ATAC-seq) data through the construction of transcription factor interaction networks (TFINs). TFINs were constructed at different timepoints using a uniform procedure. For each timepoint, we extracted all MACS2-identified ATAC peaks. For each peak, we annotate it using BEDTools to select peaks within a high-likelihood *cis*-regulatory region around each transcription factor. We select all peaks contained within the gene body (exons and introns) or 2 kb upstream using the GENCODE v31 (Grch38) gene annotation. All other ATAC peaks that are not contained within likely *cis*-regulatory regions are discarded.

Using motifmatchr and the JASPAR-2020 TF database, we next identify all TF binding sites in each ATAC peak with match probability *p* < 5 × 10^−5^ ^71,72^. This output a binarized matrix of all (putative) TFs binding in each open chromatin region. To facilitate comparison with our genome-scale TF CRISPR screen, we subset putative regulator TFs to those with both a JASPAR motif and in the hTF-GeCKO library (~630) and putative target TFs to those in the hTF-GeCKO library (1891). This leaves us with a master regulator-target matrix of all possible TF interactions with ~217,000 target-regulator pairings.

To create each TFIN, we filtered the master regulator-target matrix by specific expression and chromatin accessibility criteria. For the TFINs in Fig. 1, we used the following criteria: 1) The target TF must have a fold-change (increase or decrease) of at least 2-fold with a DEseq2 *p*_*adj*_ < 0.05 and a RSEM expected count of at least 10; 2) The regulator TF must be expressed at the *previous* timepoint (by Criteria #1 but must be an increase, not decrease); 3) The sum of all open chromatin (ATAC) reads for a specific regulator-target pair must exceed 50 reads at the specific timepoint and must be greater than the summed ATAC reads at the ES cell stage (this eliminates open chromatin that is maintained regardless of cell state, e.g. near essential genes that are not specific to differentiation). For the initial timepoint (12 hours), the regulator TFs were defined as *NEUROG1* and *NEUROG2*. For Criteria #1, the increase/decrease of the target TF expression was used to determine which TF interactions were activating or repressing. For those regulator-target TFs that meet this criteria, the interactions are analyzed in R and plotted using Cytoscape v 3.8^73^.

### Reporting summary

Further information on research design is available in the Nature Portfolio Reporting Summary linked to this article.

### Supplementary information

Supplementary Information

Description of Additional Supplementary Files

Supplementary Data 1 - 5

Reporting Summary

### Source data

Source Data

## Data Availability

The data supporting the findings of this study are available within the paper and its supplementary information files. All datasets (CRISPR screen, RNA-sequencing and ATAC-sequencing) have been deposited in the NCBI BioProject repository (PRJNA1002468). The following publicly available datasets have also been used in the study: BrainSpan atlas [https://www.brainspan.org/]; ZBTB18 chromatin immunoprecipitation (ChIP-seq) dataset GEO dataset GSM2026861. Source data are provided with this paper.
