# Supplementary material for: Essential transcription factors for induced neuron differentiation
Source: Nat Commun. 2023 Dec 15;14:8362. doi: 10.1038/s41467-023-43602-7 (PMC10724217; doi:10.1038/s41467-023-43602-7)
Supplement: Supplementary file 1 — Supplementary Information [file 41467_2023_43602_MOESM1_ESM.pdf]

Supplementary materials

Essential transcription factors for induced neuron differentiation

Congyi Lu[1,2,*], Görkem Garipler[2,*], Chao Dai[1,2,*], Timothy Roush[1,2], Jose Salome-Correa[1,2], Alex Martin[1,2], Noa Liscovitch-Brauer[1,2], Esteban O. Mazzoni[2, ‡,†], Neville E. Sanjana[1,2,†]

1. New York Genome Center, New York, NY, USA
2. Department of Biology, New York University, New York, NY, USA

[*] Equal contribution.

[‡] Present address: Department of Cell Biology, NYU Grossman School of Medicine, New York, NY USA

[†] Corresponding author: eom204@nyu.edu, nsanjana@nyu.edu

Supplementary Figures

Supplementary Fig. 1. A diverse set of transcription factors are expressed during neuronal differentiation.

Supplementary Fig. 2. Fluorescence activated cell sorting (FACS) gating strategy for MAP2-tdTomato reporter CRISPR screen.

Supplementary Fig. 3. Early time point guide RNA depletion and correlation between different induced neuron populations.

Supplementary Fig. 4. Gene expression and target site accessibility of neuron-essential transcription factors (neTFs).

Supplementary Fig. 5. Enhanced accessibility of the promoter region of *NEUROG2, POU3F2, VAX2* and *ZBTB18* upon activation of *NEUROG1/2*.

Supplementary Fig. 6. A mouse TFome-scale CRISPR screen in pluripotent cells to identify required TFs for *NEUROG1/2*-induced neuronal differentiation.

Supplementary Fig. 7. ZBTB18 expression during fetal development and genotyping of ZBTB18-null isogenic cell lines.

Supplementary Fig. 8. Gene expression and chromatin accessibility analyses in ZBTB18-null isogenic cell lines.

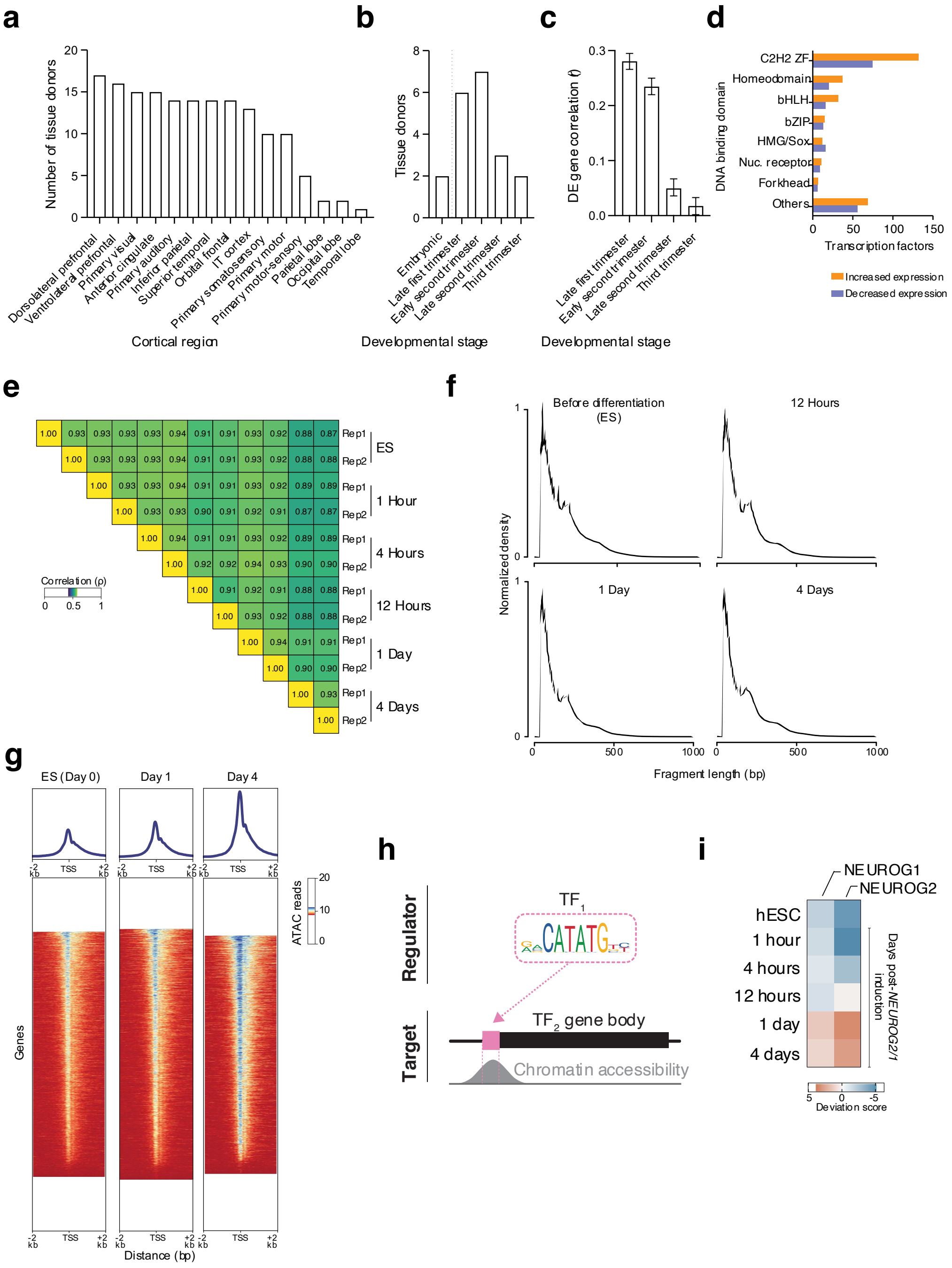

Supplementary Figure 1. A diverse set of transcription factors are expressed during neuronal differentiation. (a) Human fetal RNA-sequencing cortical regions used from the Allen Institute BrainSpan Developing Brain atlas. A total of 20 donors were analyzed with gene expression values averaged across all cortical regions available for each donor. **(b)** Human fetal RNA-sequencing donors by developmental stages. Embryonic: 3-9 weeks post-conception (wpc). Late first trimester: 10- 13 wpc. Early second trimester: 14 - 21 wpc. Late second trimester: 22 - 26 wpc.

Third trimester: more than 26 wpc. **(c)** Pearson correlation between differentially-expressed genes in differentiated human neurons (Day 7 vs. embryonic stem cells) and differentially-expressed genes in BrainSpan human fetal cortical regions (developmental stage indicated on the *x*-axis vs. cortical tissue from embryonic stage [3-9 wpc] donors). Bars indicate Pearson correlation (*r*) with 95% confidence interval error bars. **(d)** Transcription factors with a significant change (FDR < 0.05, $|\log_2(\text{fold-change})| > 1$) in differential gene expression (Day 7 vs. ES) by DNA binding domain. **(e)** Spearman correlation between biological replicate ATAC-seq samples at different timepoints during iN differentiation (ES, 1 hour, 4 hour, 12 hour, 1 day and 4 day). **(f)** Sample insert lengths in ATAC-seq libraries display nucleosomal banding patterns. **(g)** ATAC-seq signal centered on 5000 gene promoters in Day 4 neurons. Each column is a separate biological replicate. **(h)** Motif identification strategy in the *cis*-regulatory region for each transcription factor to identify regulator-target interactions. Regulators motif were examined in the gene body (exons and introns) plus 2 kb upstream of the transcription start-site. **(i)** Accessibility of *NEUROG1* and *NEUROG2* motifs genome-wide at different timepoints using ChromVar analysis of ATAC-seq datasets [71].

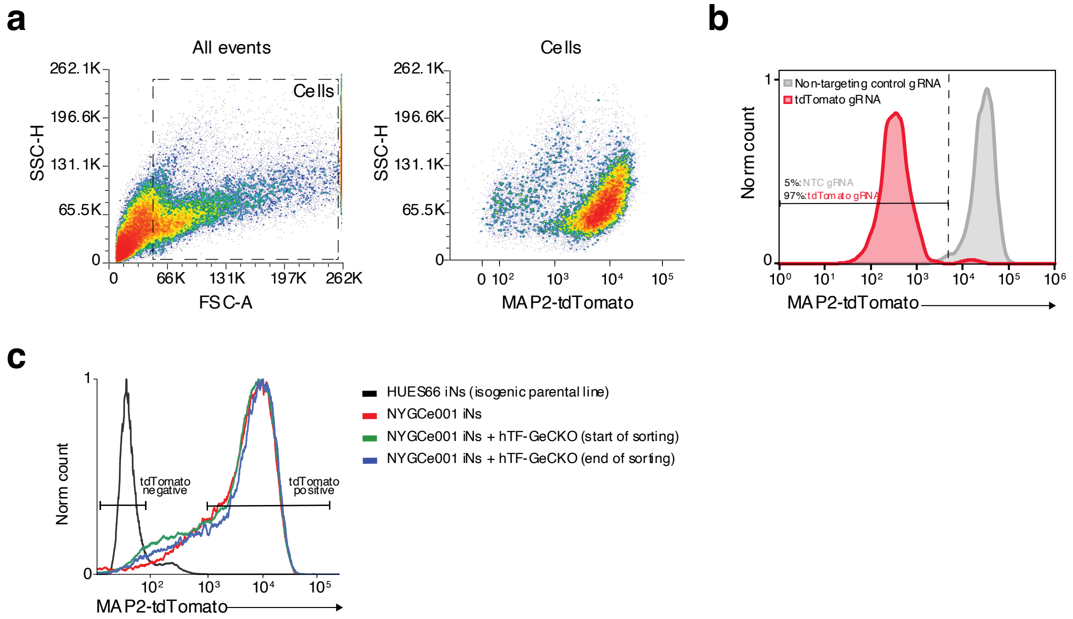

Supplementary Figure 2. Fluorescence activated cell sorting (FACS) gating strategy for MAP2-tdTomato reporter CRISPR screen. (a) Gating for cells after *NEUROG1/2* induction. **(b)** MAP2-tdTomato fluorescence in *NEUROG1/2*-induced cells transduced with either a tdTomato-targeting or a non-targeting control (NTC) guide RNA. **(c)** MAP2-tdTomato fluorescence in *NEUROG1/2*-induced cells differentiated from HUES66, NYGCe001, or NYGCe001 transduced with the hTF-GeCKO library. For the NYGCe001 transduced with the hTF-GeCKO library, the population is shown at the beginning and at the end of the multi-hour sorting period. Gates for tdTomato-positive and tdTomato-negative populations used in the pooled CRISPR screen are shown.

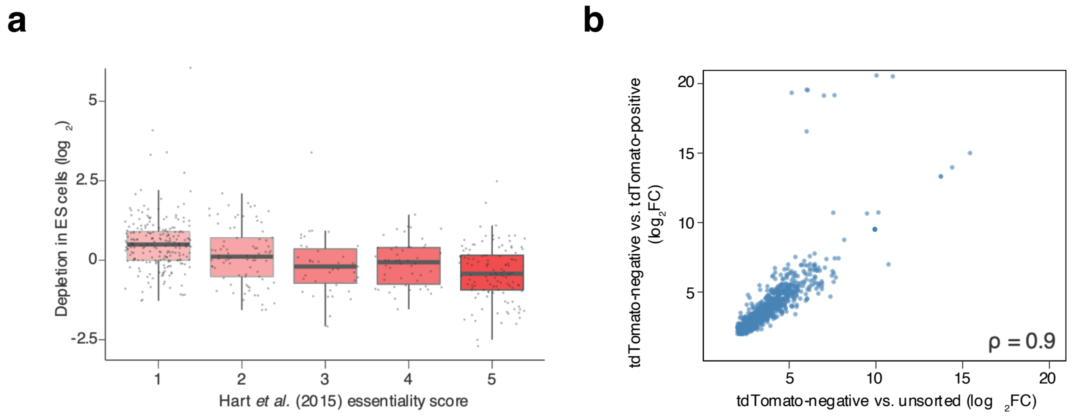

Supplementary Figure 3. Early time point guide RNA depletion and correlation between different induced neuron populations. (a) Depletion in NYGCe001 human embryonic stem cells at Day 7 of genes previously found to be essential in CRISPR screens [73]. The *x*-axis value indicates the number of cell lines where each gene was classified as essential in the Hart *et al*. dataset. **(b)** Correlation of guide RNAs in the tdTomato-negative vs. tdTomato-positive contrast and the tdTomato-negative vs. unsorted contrast.

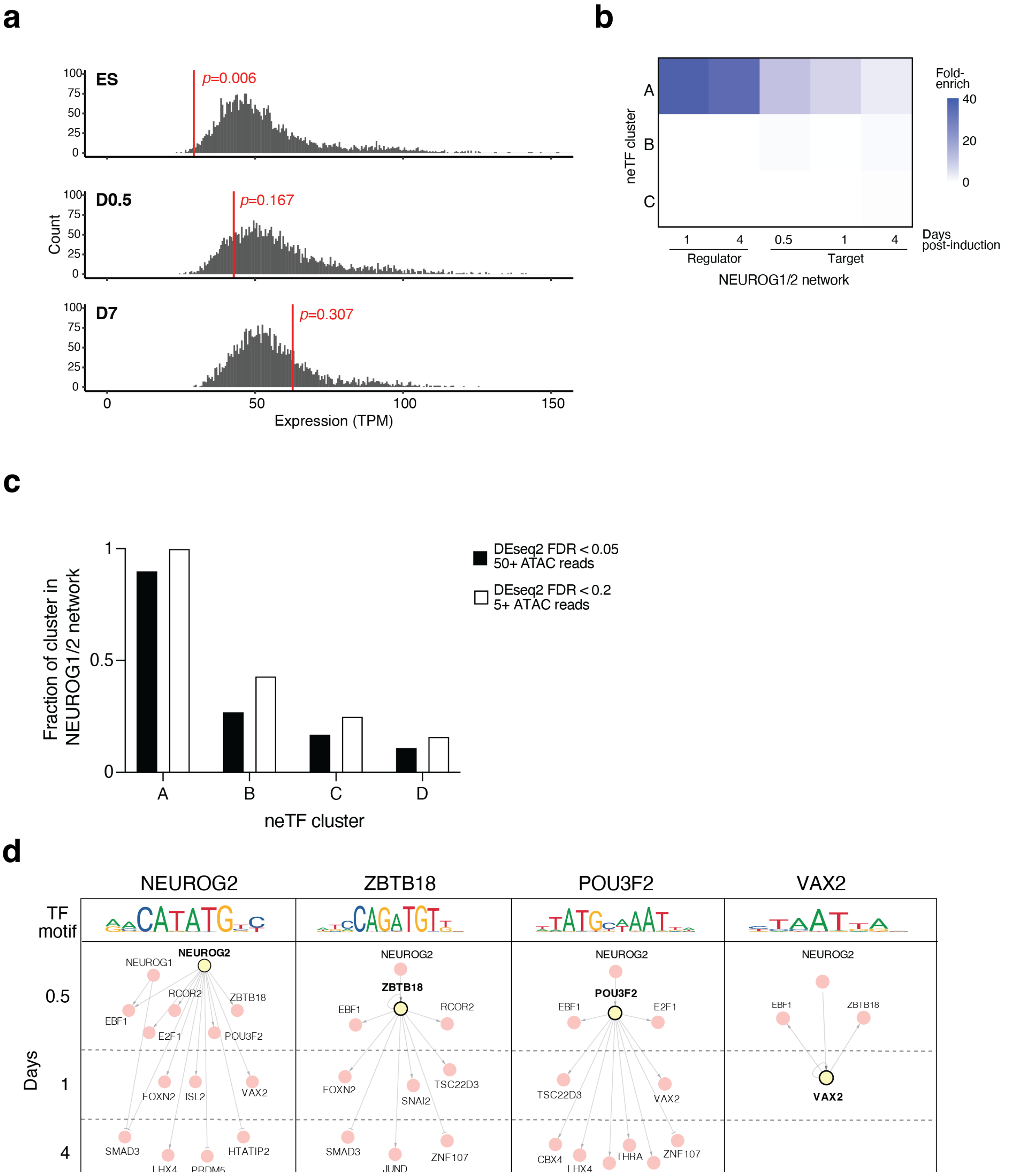

Supplementary Figure 4. Gene expression and target site accessibility of neuron-essential transcription factors (neTFs). (a) Histogram of TF expression at 3 different timepoints before or during NEUROG1/2-induced differentiation. Only expressed TFs are shown (RSEM count > 10). The mean expression of the 120 neTFs at 3 different timepoints before or during *NEUROG1/2*-induced differentiation (red line) is superimposed on each graph. We indicate the empirical probability of a randomly-chosen set of expressed TFs (n = 10,000 random sets) having the same

mean expression as the neTFs. **(b)** Fold-enrichment for cluster A, B and C neTFs in regulator-TF or target-TF sets from *Figure 1D*. **(c)** Fraction of neTFs by cluster that are present in the NEUROG1/2 network as either a regulator-TF or target-TF using strict or relaxed criteria for TF network inclusion. For the networks shown in *Figure 1D*, we require target TFs to be differentially expressed (vs. stem cells) with a DEseq2 FDR < 0.05 and regulator TFs of those target TFs to have their motifs in open chromatin regions with at least 50 ATAC reads ("strict criteria network"). Using more relaxed criteria (DEseq2 FDR < 0.2, ATAC reads > 5), the resulting TF network is considerably more connected (575 nodes vs. 458 nodes in the strict criteria network, 8658 edges vs. 5517 edges in the strict criteria network). **(d)** The connections of NEUROG2, ZBTB18, POU3F2 (BRN2) and VAX2 to other neTFs.

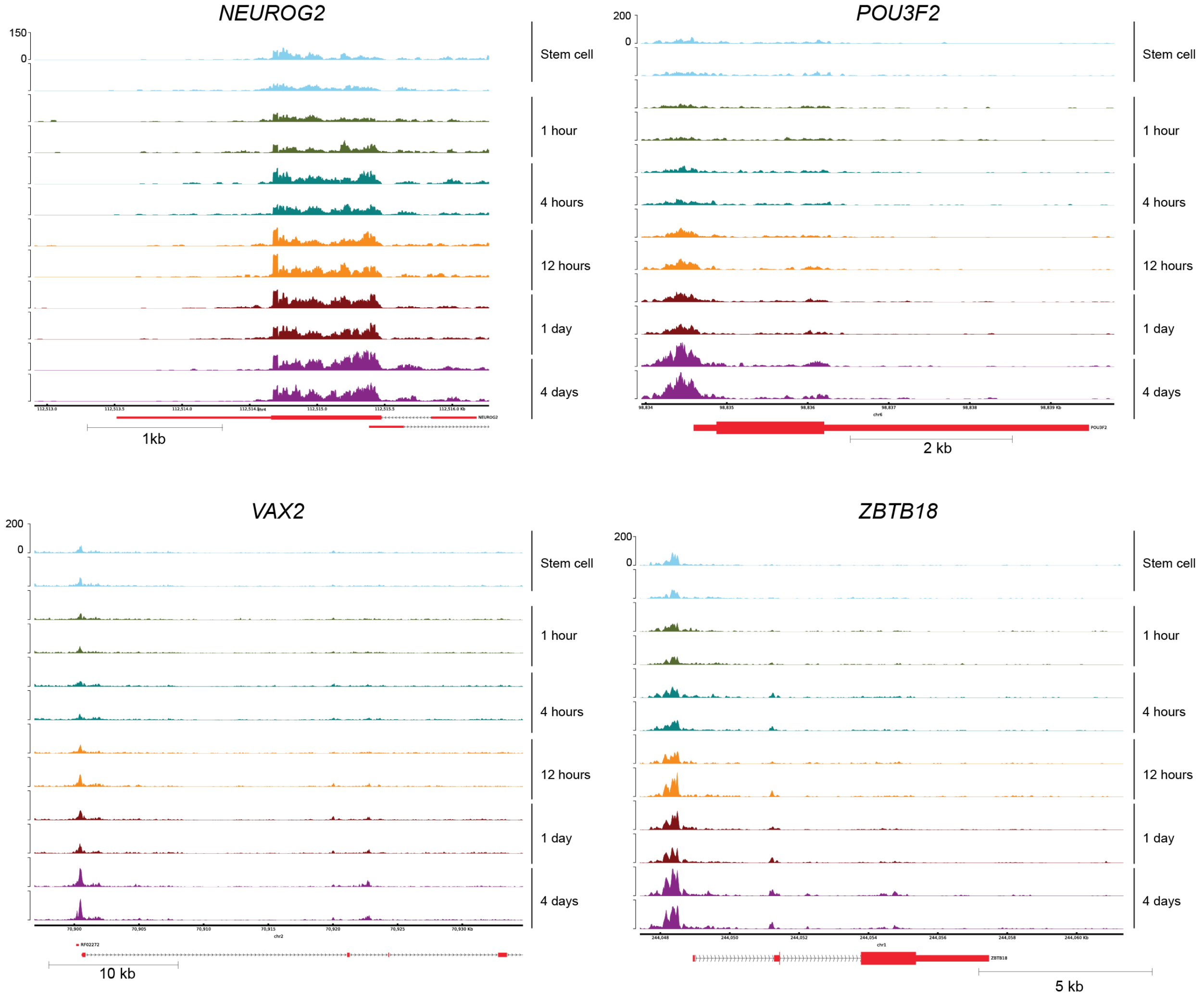

Supplementary Figure 5. Enhanced accessibility of the promoter region of *NEUROG2, POU3F2, VAX2* and *ZBTB18* upon activation of *NEUROG1/2*. ATAC reads mapping to human genome at 6 different timepoints before or during *NEUROG1/2*-induced differentiation shows enhanced accessibility of the promoter region of *NEUROG2*, *POU3F2*, *VAX2* and *ZBTB18* upon activation of *NEUROG1/2*. Two biological replicate ATAC-seq libraries are shown at each timepoint and for each track the *y*-axis is as indicated in the top track of the gene.

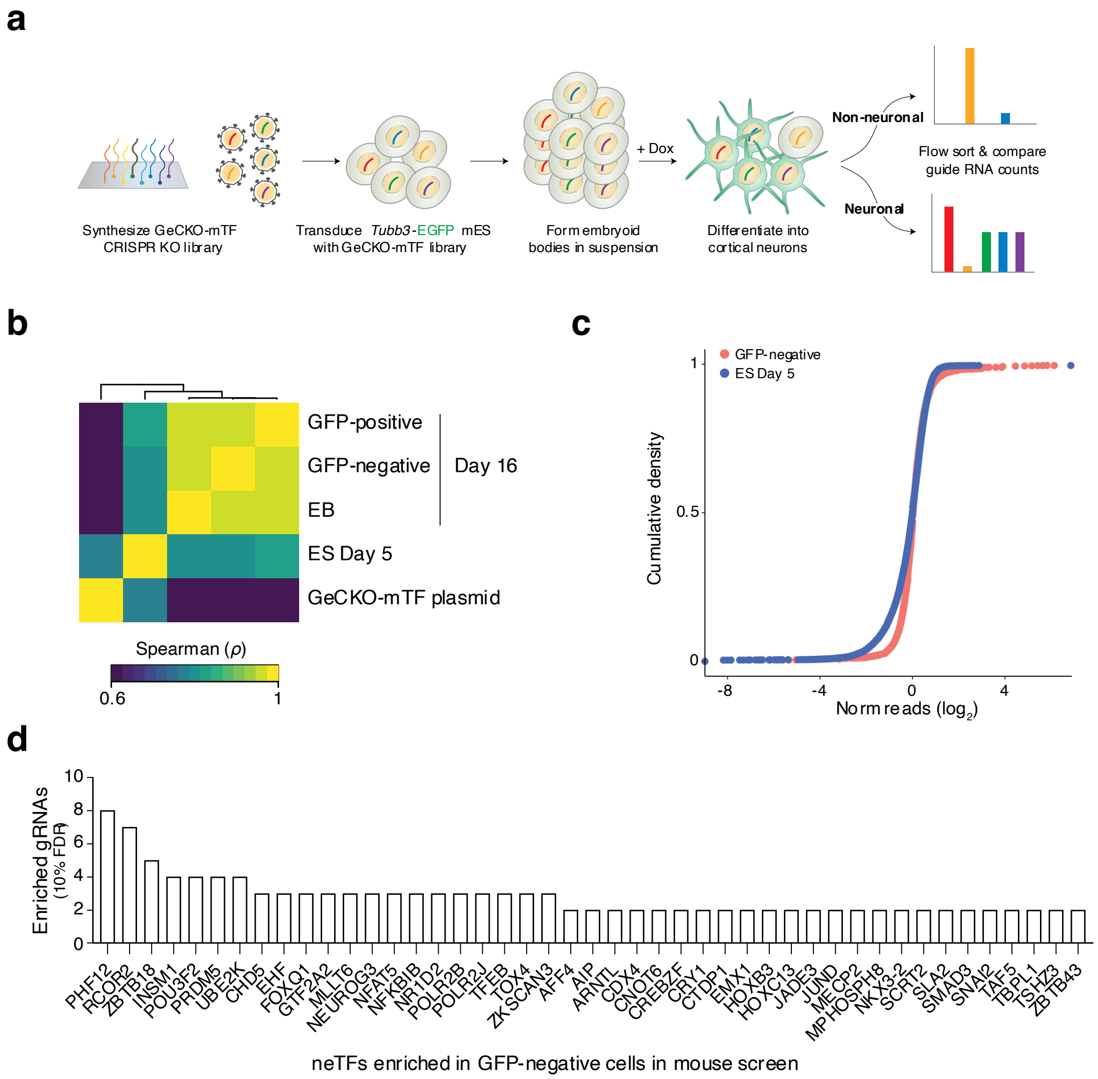

Supplementary Figure 6. A mouse TFome-scale CRISPR screen in pluripotent cells to identify required TFs for NEUROG2-induced neuronal differentiation. (a) Schematic of CRISPR screen targeting 1,682 mouse TFs with 10 guide RNAs each. **(b)** Spearman correlation between normalized read counts for each sample. ES: embryonic stem cell at early time point (Day 5). EB: embryoid body before sorting (Day 16). GFP-positive: EB sample after sorting for TUBB3-GFP-positive cells. GFP-negative: EB sample after sorting for TUBB3-GFP-negative cells. **(c)** Guide RNA representation for the ES cells and TUBB3-GFP-positive sorted populations. **(d)** Enriched guide RNAs for mouse neuron-essential TFs (enriched in the TUBB3-GFP-negative population, FDR < 0.1) that overlap neuron-essential TFs from the human CRISPR screen (*Figure 2*).

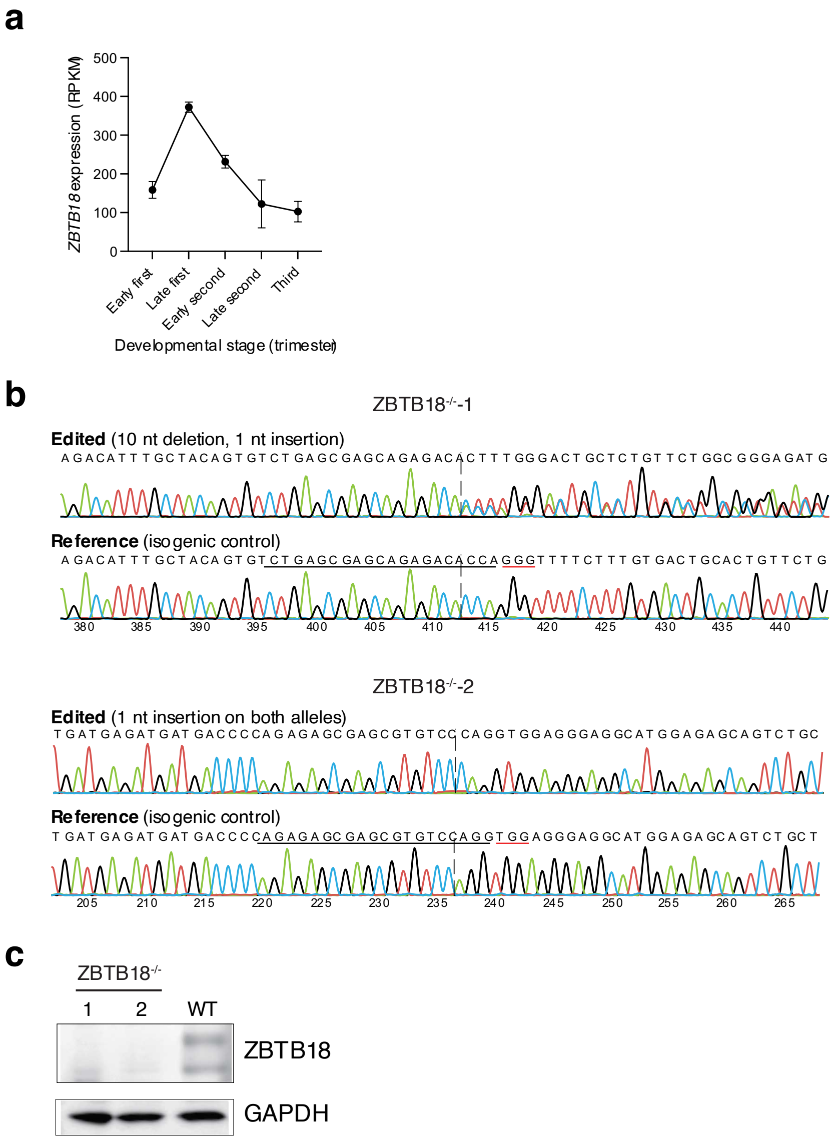

Supplementary Figure 7. ZBTB18 expression during fetal development and genotyping of ZBTB18-null isogenic cell lines. (a) *ZBTB18* expression in human fetal brain by developmental stage using RNA-sequencing from the Allen Institute BrainSpan Developing Brain Atlas. Early first trimester (embryonic): 3-9 weeks post-conception (wpc). Late first trimester: 10- 13 wpc. Early second trimester: 14 - 21 wpc. Late second trimester: 22 - 26 wpc. Third trimester: more than 26 wpc. Mean and standard error of *ZBTB18* expression in reads per kilobase per million (RPKM). For all donors, RPKM values are first averaged over all cortical regions (see *Figure S1A*). **(b)** Sanger sequencing of the ZBTB18-null cell lines (1 and 2) engineered using 2 different

guide RNAs. **(c)** Western blot analysis of ZBTB18-null cell lines (and isogenic parental cell line, WT) using a validated rabbit polyclonal ZBTB18 antibody [30,31,74].

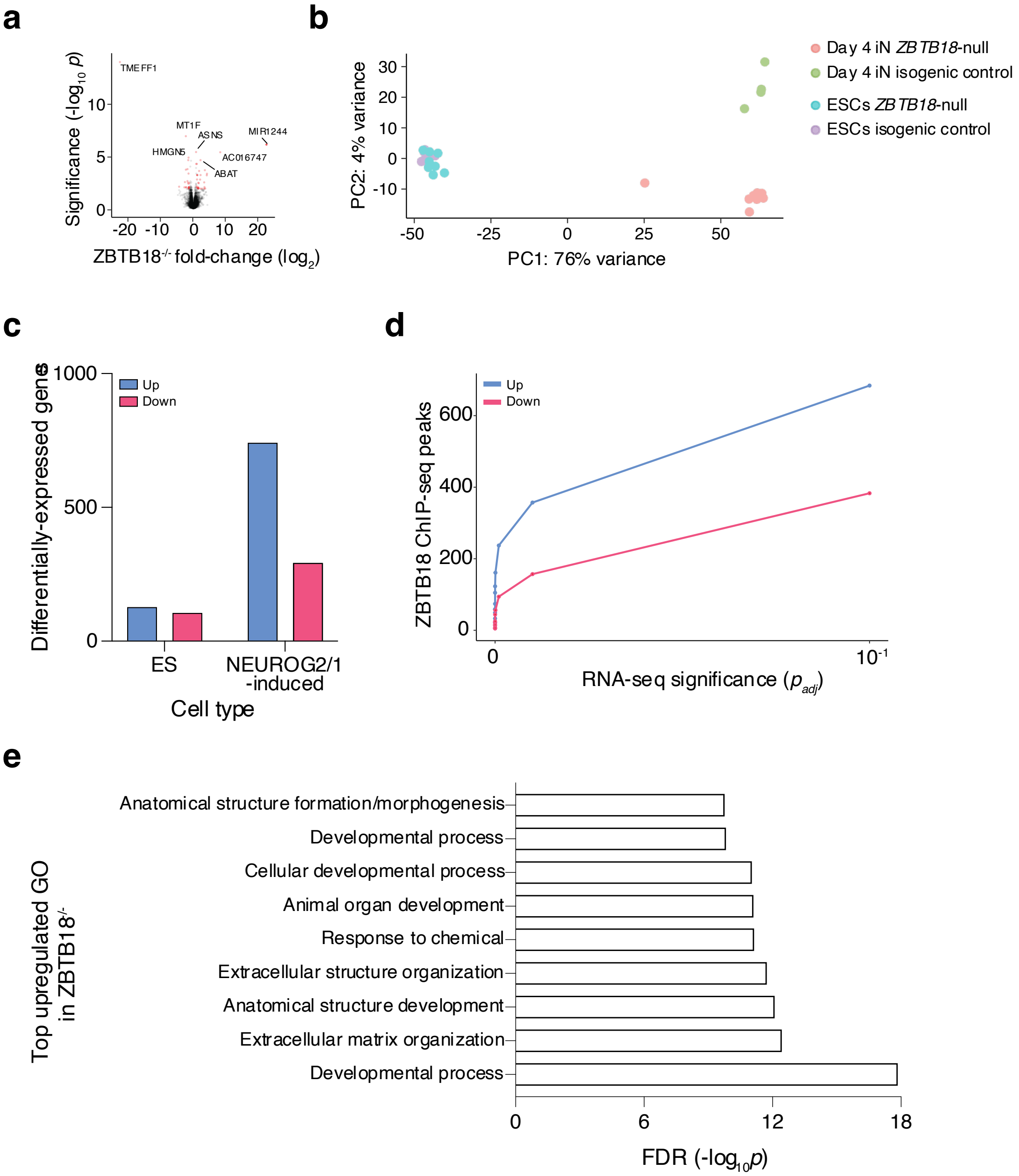

Supplementary Figure 8. Gene expression and chromatin accessibility analyses in ZBTB18-null isogenic cell lines. (a) Volcano plots for differential gene expression of ZBTB18-null pluripotent stem cells (n = 4 replicates per genotype and timepoint). Red dots indicate genes with $p_{adj} < 0.01$ and |fold-change| > 2. **(b)** Principal components analysis of gene expression in ZBTB18-null and parental isogenic (wild-type) control cell lines. Although the major source of variance (PC1) is cell state (pluripotent stem cell vs. induced neuron), PC2 captures a difference between genotypes (ZBTB18-null vs. wild-type) that is only found in the *NEUROG1/2*-induced cells and not in the pluripotent stem cells. **(c)** Number of differentially expressed genes ($p_{adj} < 0.1$ and |fold-

change| > 2) in ZBTB18-null and parental isogenic (wild-type) control cell lines in pluripotent stem cells and *NEUROG1/2*-induced cells. **(d)** Number of ZBTB18 ChIP-seq peaks where the closest gene is a significantly differentially expressed genes in ZBTB18-null vs. parental isogenic (wild-type) control cell lines. Data is shown for multiple gene expression (DEseq2) significance cut-offs from 10^{-14} to 10^{-1}. ZBTB18 ChIP-seq data is from GEO dataset GSM2026861 [32]. Up indicates genes with increased expression in ZBTB18-null differentiated cells and down indicates decreased expression in ZBTB18-null differentiated cells. **(e)** Top-ranked Gene Ontology categories in significantly upregulated genes in ZBTB18-null differentiated cells at day 4 after NEUROG1/2-induction.
