# Supplementary material for: Essential transcription factors for induced neuron differentiation
Source: Nat Commun. 2023 Dec 15;14:8362. doi: 10.1038/s41467-023-43602-7 (PMC10724217; doi:10.1038/s41467-023-43602-7)
Supplement: Supplementary file 2 — Description of Additional Supplementary Files [file 41467_2023_43602_MOESM2_ESM.pdf]

Description of Additional Supplementary Files

File Name: Supplementary Data 1
Description: RNA- and ATAC-seq library descriptions and sequencing reads. Sample and experiment type with the corresponding numbers of sequenced and mapped reads.

File Name: Supplementary Data 2
Description: Regulator and target genes. The regulator and target genes in the TF networks at different timepoints after *NEUROG1/2* induction.

File Name: Supplementary Data 3
Description: Human TF CRISPR screen normalized gRNA counts. Unique identifier and gene target for each guide RNA in the GeCKO-hTF (human) library and normalized gRNA counts from plasmid (Plasmid), hESCs at day 7 (D7_ES), *NEUROG1/2*-induced cells at day 7 (D7_neuron) and flow sorted MAP2-tdTomato positive (MAP2-pos) and negative (MAP2-neg) populations.

File Name: Supplementary Data 4
Description: Neuron-essential TFs (neTFs) and *k*-means cluster identity. Gene names and *k*-means cluster labels for the 120 neTFs.

File Name: Supplementary Data 5
Description: Mouse TF CRISPR screen normalized gRNA counts. Unique identifier and gene target for each guide RNA in the GeCKO-mTF (mouse) library and normalized gRNA counts from plasmid (Plasmid), mESCs at day 7 (ES_D5), embryoid body (EmyBody), flow sorted TUBB3-GFP positive (GFPpos), and negative (GFPneg) populations.
